# The Economic impact of ME/CFS: Individual and societal costs

**DOI:** 10.1186/1476-5918-7-6

**Published:** 2008-04-08

**Authors:** Leonard A Jason, Mary C Benton, Lisa Valentine, Abra Johnson, Susan Torres-Harding

**Affiliations:** 1 Department of Psychology, Depaul University, Center for Community Research, Chicago, IL, USA; 2 Department of Psychology, Wichita State University, Wichita, KS, USA; 3 Department of Psychology, University of North Texas, Denton, TX, USA; 4 Department of Psychology, Roosevelt University, Chicago, IL, USA

## Abstract

**Background:**

ME/CFS is characterized by debilitating fatigue in addition to other physical and cognitive symptoms. It is estimated to affect over 800,000 adults in the U.S. ME/CFS often results in diminished functionality and increased economic impact. The economic impact of an illness is generally divided into two categories: direct and indirect costs. Despite high prevalence rates and the disabling nature of the illness, few studies have examined the costs of ME/CFS at the individual and societal level. In fact, of the four studies examining the economic impact of ME/ME/CFS only two used a U. S. sample. The current study used community and tertiary samples to examine the direct costs of ME/CFS.

**Methods:**

Using archival data, Study 1 examined the direct cost of ME/CFS in a community-based sample in Chicago. Study 2 estimated the direct cost of ME/CFS in a tertiary sample in Chicago. Both Study1 and Study 2 assessed direct costs using office visit costs, medical test costs, and medication costs.

**Results:**

For Study 1, the annual direct total cost per ME/CFS patient was estimated to be $2,342, with the total annual direct cost of ME/CFS to society being approximately $2 billion. In Study 2, the annual direct was estimated to be $8,675 per ME/CFS patient, with the total annual direct cost of ME/CFS to society being approximately $7 billion.

**Conclusion:**

Using ME/CFS prevalence data of 0.42 and indirect costs estimates from Reynolds et al. (2004), the direct and indirect cost of ME/CFS to society was estimated to be $18,677,912,000 for the community sample and $23,972,300,000 for the tertiary sample. These findings indicate that whether or not individuals are recruited from a community or tertiary sample, ME/CFS imposes substantial economic costs.

## Background

According to Jason et al. [1], chronic fatigue syndrome (CFS) affects over 800,000 adults in the United States. This illness is has more recently been referred to as ME/CFS (where ME stands for either Myalgic Encephalomyelitis or Myalgic Encephalopathy). The prognosis for severely afflicted patients with ME/CFS is poor [2,3]. The persistent and debilitating nature of ME/CFS often results in a reduction in work and family life activities, as well as an increase in health care costs [4]. Because it becomes difficult for patients with ME/CFS to continue employment at premorbid levels, many have little choice but to leave their jobs. Indeed, Jason et al. [1] found that participants with ME/CFS were more likely to be receiving disability income, be unemployed, or be working part-time than control participants. Similar findings of higher unemployment rates among patients with ME/CFS were found in Bombardier and Buchwald [5]; McCrone, Darbishire, Ridsdale, and Seed [6]; Reynolds, Vernon, Bouchery, and Reeves [7] and Tiersky, DeLuca, Dhar, Jonson, and Lange [8]. In addition to employment loss, patients with ME/CFS often experience escalating costs of health care due to the search for a more definitive diagnosis and treatment [9].

The economic impact of an illness is typically examined in terms of direct and indirect costs. The former refers to direct medical costs including hospital, ambulatory, prescription medications, over-the-counter medications, and medical laboratory testing. Indirect costs include transportation, work productivity losses, disability reimbursements, loss of leisure or duties at home, or services provided by family members, friends, or other informal care providers [10]. Four studies have examined the economic impact of ME/CFS, three of which use clinic-based, or tertiary samples, and only two of those samples are from the United States. McCrone et al. [6] examined both direct and indirect costs and found a higher proportion of medical service use and unemployment among the ME/CFS group, as well as higher lost employment costs and combined service costs for patients with ME/CFS in tertiary care settings in the United Kingdom. Using an Australian tertiary sample, Lloyd and Pender [11] estimated an average cost of $9,436 per patient with ME/CFS, including about AU $2,000 per patient in direct medical costs. Extrapolating this figure to the population of Australia, Lloyd and Pender [11] estimated that ME/CFS cost the government in excess of $25 million and cost the Australian community approximately AU $59 million. Bombardier and Buchwald [5] examined the direct cost of patients with ME/CFS in the United States using patients from a referral clinic. The estimated average annual expenditure was $1,013 per ME/CFS patient. Reynolds et al. [7] used a community-based sample from Wichita, Kansas to estimate the indirect cost of ME/CFS. These authors estimated that the annual total value of lost productivity in the United States was $9.1 billion, or about $20,000 per individual with ME/CFS.

The previous studies of the economic impact of ME/CFS provide evidence of the financial burden placed on individuals and their families, as well as on society as a whole, however, none of the previous studies have estimated these costs using both community-based and tertiary samples. The purpose of Study 1 was to estimate the direct cost of ME/CFS to individuals with the illness and society using a community-based sample while Study 2 examined direct costs using a tertiary sample.

## Study 1

### Method

#### Participants

The data in the community-based study were derived from an epidemiologic study of ME/CFS that was carried out in three stages between 1995 and 1998 (for more details see Jason et al., [1]). In the first Stage, 18,675 adults representing a stratified random sample were screened for ME/CFS using a telephone survey. Of these participants, 780 of the respondents reported having six or more months of fatigue. Those participants who screened positive for ME/CFS-like illness, based on the Fukuda et al. [12] criteria and a control sample that screened negative were invited back for Stage 2 to complete a structured psychiatric interview. The Structured Clinical Interview for *DSM-IV *(SCID) was used to assess current and lifetime psychiatric diagnoses [13]. In Stage 3, a physician conducted a detailed medical examination to rule out any exclusionary medical conditions. A team of four physicians and a psychiatrist made a final diagnosis for each participant using the current US case definition of ME/CFS [12]. Of the 213 participants who completed all three Stages of the study, 47 were classified as having no fatigue, 45 were classified as having idiopathic chronic fatigue (ICF), 89 had chronic fatigued explained by a medical or psychiatric condition (CF-Explained), and 32 were diagnosed with ME/CFS.

The current study focused on the 47 no fatigue control participants and the 32 participants diagnosed with ME/CFS. During Stage 3, participants were asked to sign a medical release form and provide the names of their previous physicians, as well as a copy of their previous medical records. Our analyses relied on information in the medical records and therefore participants were excluded from the cost estimate if they did not provide complete medical record data. Twenty-one of the ME/CFS participants and 24 of the controls provided a complete set of medical records. To create a healthy comparison group, the control participants were screened for any chronic health conditions. According to participant medical records, the only exhibited chronic conditions were hypertension, diabetes, and hypothyroidism. For the purposes of a healthy control group, participants in the no fatigue group with one or more of these three illnesses were excluded from these analyses. Following these exclusions, 21 ME/CFS participants and 15 control participants were included in cost estimates for this study. Sociodemographic information is provided in Table 1. Of the final sample of 36 participants, 13.9% were African American, 55.6% were Caucasian, 22.2% were Latino and 8.3% reported other ethnicity. From this sample 61.1% were females and 38.9% were males. Age of the participants ranged from 20 to 64, with a mean age of 37.

**Table 1:** Community Sample Characteristics

	ME/CFS Group (*N *= 21)		Control Group (*N *= 15)	
	*N*	%	*N*	%
Race				
African American	3	14.3	2	13.3
Caucasian	10	47.6	10	66.7
Latino	6	28.6	2	13.3
Other	2	9.5	1	6.7
Sex				
Women	14	66.7	8	53.3
Men	7	33.3	7	46.7
Education				
Some or less than high school	2	9.5	0	0.0
High school degree or part college	9	42.9	6	40.0
Standard college degree	8	38.1	4	26.7
Graduate/professional degree	2	9.5	5	33.3
Occupation				
Unskilled worker	3	14.3	2	13.3
Skilled worker	2	9.5	1	6.7
Clerical worker	4	19.0	2	13.3
Technician	4	19.0	2	13.3
Manager	6	28.6	6	40.0
Administrator	2	9.5	2	13.3
Age				
18 – 29	7	33.3	3	20.0
30 – 39	4	19.0	7	46.7
40 – 49	7	33.3	4	26.7
50 – 59	2	9.5	1	6.7
60 – 69	1	4.8		
Socioeconomic Status Scores				
5 – 14	3	14.3	2	13.3
15 – 24	1	4.8	1	6.7
25 – 34	8	38.1	2	13.3
35 – 44	7	33.3	7	46.7
45 – 54	2	9.5	3	20.0

#### Measures

##### CFS Screening Questionnaire

The CFS Screening Questionnaire, developed by Jason, Ropacki, and Santoro [14], was administered to all participants. In addition to screening for ME/CFS symptomology, the questionnaire assessed sociodemographic characteristics including current work status and socioeconomic status variables. Specifically, participants were asked if they were receiving disability income, were unemployed, were working part-time, were working full-time, or were retired. Participants who indicated that they were currently employed were asked to provide information about their current job, including hours of employment, and preferred hours of employment, as well as about conditions in their current work place. Information from this questionnaire was used to assess work loss among participants, as well as to examine differences in the amount of time spent at work. Participants were also asked to report about their disability including the need for help from a professional to get employment accommodations, reduction in work activities, and difficulty performing work activities.

##### The Medical Questionnaire

The Medical Questionnaire, a self-report measure, is a modified version of the Chronic Fatigue Questionnaire developed by Komaroff, Faglioli, and Geiger [15]. The questionnaire was used to assess current and past medical history including information about medication practices. Participants were asked to list their current usage of medications (prescription and over-the-counter), reasons for medications, and doses they were currently taking.

##### Previous medical record

In addition to the self-report measures, participants were asked to provide a copy of previous medical records. These included the name, address, and phone number of the participants' primary care physician, office visit dates and notes, and medical test names, dates, and results. Medical records were used to assess amount of medical service use. For each participant, the most recent full year of medical record information was used in the estimates.

## Procedure

The objective of Study 1 was to estimate the economic impact of ME/CFS on an individual and at the societal level. The direct economic impact of ME/CFS was estimated using current medication, medical test and medical office visit prices. As part of the Medical Questionnaire, each participant was asked to list the medications that they were currently taking. Typical drug dosages and quantity needed for a 30-day supply were calculated using the *Physician's Desk Reference Monthly Prescribing Guide *[16]. Current drug prices were obtained using the February 2005 *Update to the RedBook: Pharamacy's Fundamental Reference *[17]. The *RedBook *provides cost information for prescription and over-the-counter medications. For each drug, the *RedBook *gives the generic name, the active ingredient, and the average wholesale price for various doses and quantities of each medication. A monthly medication cost was calculated for each participant. Each participant's monthly cost was then multiplied by twelve and the products were averaged to estimate the annual cost of medication.

Medical test usage was another direct measure of the economic cost of ME/CFS. Based on the most recent year of medical records, specific medical tests received by each participant were recorded. Current medical test prices were obtained from two public Chicago hospitals. These prices were the actual fee that the hospitals charged for the tests, not accounting for different types of medical insurance. The annual costs of medical tests were calculated using the average prices from these two hospitals.

Costs for medical office visits were also included in the direct estimate of the economic impact of ME/CFS. The number of office visits per year was calculated by counting the office visits documented in a year of medical record data for each participant. The average cost of office visits among established and new patients in the East North Central region of the U.S., published in the American Medical Association's Socioeconomic Characteristics of Medical Practice was $76.55. This cost was used to calculate the cost per participant per year for medical office visits [18].

Annual costs of medication usage, medical tests, and medical office visits were summed to calculate the average total annual direct costs of patients with ME/CFS and for comparison purposes, the control participants. This average sum of fees was considered the direct costs to individuals with ME/CFS and their families. Societal level impact was assessed by multiplying the total annual direct cost by the estimated number of adults in the United States with ME/CFS, using the prevalence rate of 0.42 published by Jason et al. [1], or approximately 836,000 adults.

## Results

### Sociodemographic variables

Data from 36 participants, 21 participants with ME/CFS and 15 control participants, were analyzed. The demographic characteristics of the community sample are detailed in Table 1. There were no significant differences between participants with ME/CFS and control participants for ethnicity, gender, education level, occupation, age, or socioeconomic status. A significantly higher proportion of participants with ME/CFS reported having to cut down on the amount of time spent on work or other related activities (M = .80) when compared to control participants (M = .33; χ^2^(2, *N = *35) = 10.02, *p *= .01). Participants with ME/CFS (70%) reported more difficulty performing work or other activities than controls (13.3%), χ^2^(2, *N *= 35) = 13.10, *p *= .00. Participants with ME/CFS were more likely to feel that they might need help from a professional in order to receive employment accommodations than controls (40% versus 0%, respectively, χ^2^(2, N = 35) = 6.20, *p *= .05). A significantly higher percentage of participants with ME/CFS than controls were receiving disability income (19% vs. 0%), unemployed (23.8% vs. 6.7%) or working part-time (19% vs. 6.7%). When current work status variables were collapsed into two categories, working full-time and not working full-time (including participants who reported working part-time, being on disability, being unemployed, or being retired), the differences between participants with ME/CFS (33.3% were working full-time) and controls (86.7% were working full-time) was significant, χ^2^(1, *N *= 36) = 10.10, *p *= .00.

### Direct medical costs

Medication usage and costs were assessed in the estimate of direct medical service use. Based on self-reported use and physician exam information, the mean number of prescription medications was 1.6 (SD = 2.1) for participants with ME/CFS and .7 (SD = .9) for control participants, however these differences were not statistically significant, *t*(34) = 1.56, *p = *.13. The mean number of over-the-counter medications was .4 (SD = .6) for participants with ME/CFS and .1 (SD = .4) for controls, and these differences were not significant, *t*(33) = 1.57, *p *= .13. The total average annual cost of prescription and over-the-counter medications was significantly higher for participants with ME/CFS ($1,159; SD = 1426) than for controls ($321; SD = 415), *t*(25) = 2.55, *p *= .02.

Medical test and medical office visit costs were also included in the direct estimate of economic impact. According to medical record information, the mean number of medical tests received by participants with ME/CFS was 3.2 (SD = 3.3) and 2.0 (SD = 2.9) for control participants. These means were not significantly different, *t*(34) = 1.16, *p *= .25. Annual medical tests on average cost participants with ME/CFS $713 (SD = 1200) and control participants $470 (SD = 856), but these differences were not statistically significant, *t*(34) = .67, *p *= .51. Based on medical record data, the mean number of office visits per year was 6.1 (SD = 3.6) for participants with ME/CFS and 4.5 (SD = 3.4) for control participants, but these differences were not statistically significant, *t*(34) = 1.42, *p *= .17. Participants with ME/CFS spent an average of $470 (SD = 274) on medical office visits, while control participants spent an average of $342 (SD = 259). These cost were not statistically significant, *t*(34) = 1.42, *p *= .17. The total annual direct costs, using the mean sum of medication, medical test, and medical office visit costs, for participants with ME/CFS was $2,342 (SD = 2174) and for controls was $1,133 (SD = 1262), which approached significance, *t*(34) = 1.93, *p *= .06.

### Total costs

This total direct cost was extrapolated to the adult population of the U.S. as a whole to estimate the direct societal cost implications. We used Jason et al.'s (1999) prevalence estimates (.42) and the US Census 2000 population estimates (836,000 adults with ME/CFS). The estimated total annual direct cost of ME/CFS to society was $1, 957,912,000 ($2,342 × 836,000) or approximately $2 billion.

## Study 2

### Method

#### Participants

For the tertiary sample, 114 individuals were recruited: 46% were referred by physicians, 34% were recruited by media (newspapers, TV, radio, etc.), and 20% stemmed from other sources (e.g., heard about the study from a friend, family member, person in the study, etc.). There were no significant demographic differences for patients recruited from these varying sources. Twenty-four additional individuals who were screened were excluded due to a variety of reasons (i.e., lifelong fatigue, less than 4 Fukuda symptoms, BMI > 45, melancholic depression or bipolar depression, alcohol or substance abuse disorder, autoimmune thyroiditis, cancer, lupus, rheumatoid arthritis). Approaches to reduce attrition included use of letters and telephone reminders of all appointments, flexibility regarding working around vacations and medical and other crises, reimbursement for transportation costs, and participant honoraria.

All participants were required to be at least 18 years of age, not pregnant, able to read and speak English, and considered to be physically capable of attending the scheduled sessions. Bedridden and wheelchair bound patients were excluded due to the practical difficulties of making appointments. Referrals to local physicians who treat ME/CFS and to support groups were offered to these individuals. After a consent form was filled out, participants were provided a thorough medical and psychiatric examination, similar to what was described above for the community based sample.

#### Measures

##### CFS Screening Questionnair

The CFS Screening Questionnaire [14] was used to screen for ME/CFS symptoms and assess sociodemographic characteristics.

##### Client Service Receipt Inventory

The Client Service Receipt Inventory (CSRI) is an instrument that measures the cost of psychiatric interventions [19]. This has been adapted for use in estimating cost information for ME/CFS [6]. As part of the CSRI, participants provided details of services they have used in the previous 3 months, including general practitioners, other primary care services; in-patient hospital care; other medical physicians; osteopaths; chiropractors; physiotherapists; and acupuncturist/homeopaths. For each of these different services participants provided details including duration of contact and reason for visits. Finally, participants estimated the amount they have paid out-of-pocket for health care relating to their fatigue.

## Procedure

### Results

#### Sociodemographic variables

Data were analyzed from the 90 of the 114 participants with ME/CFS who had full economic information. The demographic characteristics of the tertiary sample are detailed in Table 2. The majority of the sample was Caucasian and female. Seventy-six percent of participants in the tertiary sample reported having to cut down on their work and related activities. Of the respondents in this sample, 27.6% stated that they were receiving disability, 21.8% were unemployed, 26.4% were working part-time, and 25.3% were working full-time.

**Table 2:** Tertiary Sample Characteristics

	(*N *= 90)	
	*N*^*a*^	%
Race		
African American	4	4.5
Caucasian	80	89.9
Latino	4	4.5
Other	1	1.1
		
Sex		
Women	75	83.3
Men	15	16.7
		
Education		
Some or less than high school	1	1.1
High school degree or part college	25	27.8
Standard college degree	44	48.9
Graduate/professional degree	20	22.2
		
Age		
18 – 29	9	10
30 – 39	21	23.3
40 – 49	27	30
50 – 59	24	26.7
60 – 70+	9	10
		
Socioeconomic Status Scores		
5 – 14	33	41.3
15 – 24	4	5.0
25 – 34	11	13.8
35 – 44	19	23.8
45 – 54	13	16.3

^*a*^*Note*. Values not equaling 90 participants signify missing data.

#### Direct costs

Medication usage and costs were estimated for direct medical service use. Based on self-reported use and physician examination information, the mean number of prescription medications was 3.7 (SD = 3.5). The mean number of over-the-counter medications was 2.2 (SD = 2.3). The total average annual cost of prescription and over-the-counter medications for this sample was much higher than the community or control sample, $5,447 (SD = 5,051). Medical test and medical office visit costs were also included in the direct estimate of economic impact. The mean number of medical tests was 6.4 (SD = 9.5). Medical tests cost $2,999 on average (SD = 7,026), while the mean number of office visits per year was 6.3 (SD = 4.4). Office visits cost an average of $228 (SD = 333). The total annual direct cost was estimated using the mean sum of medication, medical test, and medical office visit costs. The total annual direct cost was $8,675 (SD = 8,854). This is nearly three times the cost of the community sample in Study 1.

The estimated total annual direct cost of ME/CFS to society is ($8,675 × 836,000 = $7,252,000,000) or approximately $7 billion.

## Discussion

These findings suggest that there is a high economic cost associated with ME/CFS for patients, their families, and for society as a whole. This study has provided a conservative estimate of the direct economic impact of ME/CFS, with a mean annual cost of $2,342 to $8,675 per patient. When extrapolated to the U.S., the direct cost to the American health care system is estimated to be from $1, 957,912,000 to 7,252,000,000.

In other words, the total annual direct estimated costs for the tertiary sample were nearly three times the cost of the community sample.

It is at least possible that these differences might have been due to the differing characteristics of the sample. In study 1, the majority of participants with ME/CFS were minorities, whereas in study 2, 90% were Caucasian. There were also differences in the educational attainment between the two samples. Among those with ME/CFS in study 1, only 48% had at least standard college degree, whereas among those is study 2, 71% had this level of educational attainment. Finally, the majority of those in study 1 had never been diagnosed with ME/CFS, whereas all of those participants in study 2 had this diagnosis. Very possibly, those participants with ME/CFS in study 1 had less resources to devote to medical care, and the majority did not even know that had this illness. In contrast, all of those in study 2 had a ME/CFS diagnosis, and they had possibly had more resources to invest in the medical diagnosis and treatment of this illness. These data suggest that the economic cost of ME/CFS will vary between those in community-based samples who might not have been diagnosed and those in tertiary clinics, who might have a diagnosis and more resources, and these differences may influence the societal cost of ME/CFS.

These economic losses can have a substantial long-term impact on ME/CFS patients' standard of living and quality of life. With high unemployment rates among ME/CFS patients, the direct cost of medical services could become even more problematic to individuals and families due to a loss of health insurance benefits and thus, increases in out-of-pocket medical expenses. Our estimate of the direct cost of medical expenses was $2,342 to $8,675 per ME/CFS patient. This estimate is not directly comparable to previous estimates of the direct cost of ME/CFS due to different sampling strategies. Bombardier and Buchwald [5] estimated an average annual medical expenditure of $1,031 per ME/CFS patient. Estimates from Lloyd and Pender [11] and McCrone et al. [6] used samples from Australia and the U.K. respectively, and due to different health care systems and prevalence rates, are not directly comparable with the current estimate. In addition, these studies had a number of other differences that might account for the varying cost estimates (e.g., use of different diagnostic criteria and definitions; differential levels of severity of ME/CFS symptoms and other comorbidities).

In addition to the direct medical costs imposed on individuals and society, there is also a substantial economic costs related to lost productivity. Participants with ME/CFS were receiving more disability benefits, and were more likely to be unemployed or working part-time than their control counterparts. Indirect costs to the individual and society can be estimated according to a study conducted by Reynolds et al., [7]. According to their study, approximately one third of patients with ME/CFS, who in other circumstances would have participated in the work force, stopped working and for those who continued working, their income was cut by a third. This change in employment status represented an estimated annual loss of $20,000.

Although our data did not include salary variables and indirect costs could only be examined in terms of work status change and increased disability, we could apply the Reynolds [7] figure ($20,000) to our sample. For study 1, using estimates from Reynolds et al. [7], we could estimate that the annual indirect cost to society to be $16,720,000,000 (836,000 × $20,000) or almost $17 billion. Together the total indirect and direct costs to society could be estimated to be $18,677,912,000, or over 18 and a half billion dollars. For study 2, the estimated total annual indirect cost to society is 836,000 × $20,000 = $16,720,000,000. Therefore, in study 2, together the total indirect and direct costs to society equals $23,972,300,000 or close to 24 billion dollars. For studies 1 and 2, the total direct and indirect costs due to ME/CFS were estimated to range from 17 to 24 billion dollars.

When interpreting the findings from the current study, some limitations should be considered. First, our study relied on archival data, and therefore did not include some information that would have been helpful in estimating the economic impact. For example, we did not have an estimate of salary or a measure of the actual number of work hours lost. According to McCrone et al. [6], an important variable in estimating the indirect cost is the role of informal care providers. Informal care providers refer to friends or relatives that help care for the patient without remuneration, but still incur an opportunity cost. Our archival data did not include variables to estimate these types of financial losses. Therefore, we could not calculate costs due to lost productivity, and estimating a specific indirect cost was impossible. Another limitation to the archival data is that participants in the community based sample provided their medical records and completed the self-report questionnaires between 1995 and 1997. Participants infrequently listed alternative medications (herbs or supplements) or treatments (acupuncture) that are more commonly used now, but likely were not frequently used 10 years ago. Participant medical record information also did not include information about hospital stays or ambulatory use. Therefore, our estimates of the direct costs of ME/CFS are likely an underestimate because they do not include these types of service use.

In addition, current medication usage information relied on participant recall of service use, and therefore may not be accurate. However, corroboration of participant medication use was obtained by the examining physician for more than three quarters of the participants in Study 1. Also, other means of data collection besides self-report data were analyzed. The use of medical records likely provided an accurate indication of number of office visits and medical test usage. However, medical records were not available for participants in Study 2. These limitations should be addressed in future research. Ideally, estimates of the economic impact of ME/CFS should include estimates of both direct and indirect costs.

This study suggests that the direct costs of this illness are incurred variously, both by the individual patient and by society. For the community group, the prices of prescriptions and medical services and tests are calculated, but we were not able to determine what portion was covered by insurance and what part was paid out-of pocket. Furthermore, comparison to the control group shows that while the ME/CFS group spends more the total annual direct costs (the mean sum of medication, medical test, and medical office visit costs; $2,342 versus $1,133), this difference only approached significance at the .06 level. More research is clearly needed to be able to answer whether health insurance premiums are more costly on average to those with ME/CFS. It is certainly possible that the direct costs to individuals, primarily deductibles, might be less than estimated. It is also possible that there might be added costs of this illness to the medical industry, but more information is needed concerning retail prices-per-unit in order to calculate industry costs.

In conclusion, Jason et al. [1] estimates that more than 800,000 adults in the United States have ME/CFS. This figure combined with cost information reported in the current study suggests that ME/CFS has substantial economic costs, whether one uses samples recruited from the community or from a tertiary care clinic. These cost estimates in combination with high prevalence rates are some of the reasons that more research into the cause, effective diagnosis, and treatment are necessary.

## References

[B1] Jason LA, Richman JA, Rademaker AW, Jordan KM, Plioplys AV, Taylor RR (1999). A community-based study of chronic fatigue syndrome. Archives of Internal Medicine.

[B2] Hill NF, Tiersky LA, Scavalla VR, Lavietes M, Natelson BH (1999). Natural history of severe chronic fatigue syndrome. Journal of Archives of Physical Medicine and Rehabilitation.

[B3] Reyes M, Dobbins JG, Nisenbaum R, Subedar NS, Randall B, Reeves WC (1999). Chronic fatigue syndrome progression and self-defined recovery: evidence from the CDC surveillance system. Journal of Chronic Fatigue Syndrome.

[B4] Jason LA, Fennell PA, Taylor RR, (Eds) (2003). The Handbook of chronic fatigue syndrome.

[B5] Bombardier CH, Buchwald D (1996). Chronic fatigue, chronic fatigue syndrome, and fibromyalgia: Disability and health care use. Medical Care.

[B6] McCrone P, Darbishire L, Ridsdale L, Seed P (2003). The economic cost of chronic fatigue and chronic fatigue syndrome in UK primary care. Psychological Medicine.

[B7] Reynolds KJ, Vernon SD, Bouchery E, Reeves WC (2004). The economic impact of chronic fatigue syndrome. Cost Effectiveness and Resource Allocation.

[B8] Tiersky LA, DeLuca J, Hill N, Dhar SK, Jonson SK, Lange G (2001). Longitudinal assessment of neuropsychological functioning, psychiatric status, functional disability and employment status in chronic fatigue syndrome. Applied Neuropsychology.

[B9] Friedberg F, Jason LA (1998). Understanding chronic fatigue syndrome: Anempirical guide to assessment and treatment.

[B10] Robinson RL, Jones ML (2006). In search of pharmacoeconomic evaluations for fibromyalgia treatments: A review. Expert Opinion on Pharmacotherapy.

[B11] Lloyd AR, Pender H (1992). The economic impact of chronic fatigue syndrome. The Medical Journal of Australia.

[B12] Fukuda K, Straus SE, Hickie I, Sharpe MC, Dobbins KG, Komaroff A (1994). The chronic fatigue syndrome: a comprehensive approach to its definition and study. Annals of Internal Medicine.

[B13] Spitzer RL, Williams JB, Gibbon M, First MB (1995). Structured Clinical Interview of DSM-IV-Non-Patient Edition (SCID-NP, Version 20).

[B14] Jason LA, Ropacki MT, Santoro NB (1997). A screening scale for chronic fatigue syndrome: Reliability and validity. Journal of Chronic Fatigue Syndrome.

[B15] Komaroff AL, Fagioli LR, Geiger AM (1996). An examination of the working case definitions of chronic fatigue syndrome. American Journal of Medicine.

[B16] Chernin T, (Ed) (2004). Physicians desk reference: Monthly prescribing guide September, 2004.

[B17] Hogge J, (Ed) (2005). RedBook: Pharmacy's fundamental reference, February, 2005 Update.

[B18] Gonzalez ML, Zhang P, (Eds) (1998). Socioeconomic characteristics of medical practice 1997/1998.

[B19] Beecham J, Knapp M, Thornicroft G (2001). Costing psychiatric interventions. Measuring mental health needs.

